# Silent pandemic in intensive care units: Post-pandemic rise of extensively drug-resistant Acinetobacter baumannii and Klebsiella pneumoniae in ventilator-associated pneumonia 

**DOI:** 10.5578/tt.2025011064

**Published:** 2025-03-24

**Authors:** Buğra KERGET, Ferhan KERGET, Kadir ÇELİK, Gamze KOÇ

**Affiliations:** 1 Department of Pulmonary Diseases, Atatürk University Faculty of Medicine, Erzurum, Türkiye; 2 Clinic of Infection Diseases and Clinical Microbiology, Erzurum City Hospital, Erzurum, Türkiye; 3 Department of Fundamentals of Nursing, Atatürk University Faculty of Nursing, Erzurum, Türkiye

## Abstract

**ABSTRACT**

** Silent pandemic in intensive care units: Post-pandemic rise of extensively drug-resistant Acinetobacter baumannii and Klebsiella pneumoniae in ventilator-associated pneumonia **

**Introduction:** *In the post coronavirus disease-2019 era, as the pandemic’s impact diminishes, the state of our intensive care units (ICUs) remains as crucial as the well-being of individuals. While numerous studies have explored the pandemic’s effects on patients, our focus is to examine its impact on ICUs. *

**Materials and Methods:** *A total of 72 patients who were admitted to the chest diseases ICU due to hypercapnic or hypoxic respiratory failure between October 2018-April 2020 and December 2022-December 2024 and who developed ventilator-associated pneumonia during their follow-up were included in our study *

**Results:** *While Klebsiella pneumoniae and Acinetobacter baumannii cogrowth was observed in 4 of 30 patients (13.3%) pre-pandemic, it increased to 16 of 42 patients (38.1%) post-pandemic. Extensively drug-resistant (XDR) cases rose from 6 (20%) pre-pandemic to 34 (81%) post-pandemic (p < 0.001). A significant post-pandemic decline in carbapenem and beta-lactam susceptibility was noted (p < 0.001 for all). Although susceptibility to ceftazidime-avibactam, the most effective antibiotic for K. pneumoniae, decreased, the change was not statistically significant (p= 0.09). Multivariate regression analysis identified advanced age, coronary artery disease, low ejection fraction, and XDR resistance as factors increasing mortality (p= 0.03, 0.04, 0.04, 0.001, respectively). *

**Conclusion:** *During the pandemic, our ICU, where patients were treated with broad-spectrum antibiotics for a long time, has cured many patients but could not prevent the development of multi-drug resistance and XDR Acinetobacter and Klebsiella. Failure to take the necessary precautions will cause significant effects of the silent pandemic.
*

**Key words:** *COVID-19; multi-drug resistance; extensively-drug resistant; gram negative bacteria *

**ÖZ**

** Yoğun bakım ünitelerinde sessiz pandemi: Ventilatörle ilişkili pnömonide yaygın ilaç dirençli Acinetobacter baumannii ve Klebsiella pneumoniae’nin pandemi sonrası yükselişi **

**Giriş:** *Koronavirüs hastalığı-2019 pandemisinin etkilerinin geride bırakıldığı günümüzde, insanlar kadar bu pandemiden etkilenen yoğun bakımların durumu da önemli bir konudur. Pandeminin hastalar üzerindeki etkileri binlerce çalışmayla incelenmişken, biz de yoğun bakım ünitemize olan etkisini değerlendirmeyi hedefledik.
*

**Materyal ve Metod:** *Çalışmamıza, Ekim 2018-Nisan 2020 ve Aralık 2022-Aralık 2024 tarihleri arasında, hiperkapnik veya hipoksik solunum yetmezliği nedeniyle göğüs hastalıkları yoğun bakımına yatırılan ve takiplerinde ventilatör ilişkili pnömoni gelişen 72 hasta dahil edildi. *

**Bulgular:** *Pandemi öncesinde, Klebsiella pneumoniae ve Acinetobacter baumannii birlikte üremesi 30 hastanın 4 (%13.3)’ünde gözlenirken, pandemi sonrasında 42 hastanın 16 (%38.1)’sında gözlendi. Yaygın ilaç direnci (YİD) vakaları pandemi öncesinde 6 (%20) iken, pandemi sonrasında 34 (%81)’e yükseldi (p < 0.001). Pandemi sonrasında karbapenem ve beta-laktam duyarlılığında anlamlı bir düşüş kaydedildi (tümü için p < 0.001). K. pneumoniae için en etkili antibiyotik olan seftazidim-avibaktama karşı duyarlılık azalsa da bu değişim istatistiksel olarak anlamlı değildi (p= 0.09). Çok değişkenli regresyon analizine göre, ileri yaş, koroner arter hastalığı, düşük ejeksiyon fraksiyonu ve YİD direnci, mortaliteyi arttıran faktörler olarak belirlendi (sırasıyla p= 0.03, 0.04, 0.04, 0.001). *

**Sonuç:** *Pandemi sürecinde, hastaların uzun süre geniş spektrumlu antibiyotiklerle tedavi edildiği yoğun bakım ünitemiz birçok hastayı iyileştirdi, ancak çoklu ilaç direnci ve YİD Acinetobacter ile Klebsiella’nın gelişimini önleyemedi. Gerekli önlemlerin alınmaması, bu sessiz pandeminin önemli sonuçlar doğurmasına neden olacaktır. *

**Anahtar kelimeler:** *COVID-19; çoklu ilaç direnci; yaygın ilaç direnci; gram negatif bakteriler
*

## INTRODUCTION

Hospital-acquired pneumonia (HAP) continues to be a significant cause of morbidity and mortality despite advances in antimicrobial therapy and supportive care. Pneumonia that develops 48 hours after hospi- tal admission or within 48 hours after extubation in intubated patients without a prior diagnosis of pneu- monia is defined as ventilator-associated pneumonia (VAP) (1). Early and appropriate microbiological sampling is crucial for accurate treatment planning in patients with VAP (2).

It has been observed that the number of patients developing HAP has increased since the coronavirus disease-2019 (COVID-19) pandemic (3). Studies sug- gest that one of the main reasons for this rise is the redeployment of healthcare personnel without prior intensive care experience to manage the overwhelm- ing number of cases. Additionally, the frequent and often inappropriate use of antibiotics in intensive care units (ICUs) during the pandemic has significantly contributed to the development of antimicrobial resistance and multidrug-resistant infections (4). Among Gram-negative bacteria, *Klebsiella pneumo- niae, Acinetobacter baumannii*, and *Pseudomonas aeruginosa* have exhibited a concerning tendency toward antimicrobial resistance, with reported resist- ance rates reaching up to 53% in some studies (5).

*A. baumannii* remains one of the most frequently iso- lated Gram-negative pathogens in VAP cases. However, in recent years, a notable increase in multidrug-resist- ant *K. pneumoniae* co-infections has been observed*,* particularly with the emergence of carbapenem-resist- ant strains (6). Polymyxins have been among the few remaining treatment options for carbapenem-resistant

*K. pneumoniae* and *Acinetobacter* infections. However, the widespread use of polymyxins in treat- ing both pathogens has led to the alarming emergence of polymyxin-resistant strains, further complicating treatment strategies (2). Given the limited availability of new antimicrobial agents, utilizing the synergistic effects of combination antibiotic therapy remains one of the few viable options for clinicians (7).

The rising trend of antimicrobial resistance, prolonged hospital stays, and associated morbidity and mortality has posed serious challenges for both patient outcomes and the future of antimicrobial stewardship. Studies indicate that this worsening trend has accelerated following the pandemic, largely due to the increased and often indiscriminate use of antimicrobial agents.

Our study aims to compare the factors influencing antimicrobial resistance and mortality in VAP patients infected with *Acinetobacter* and/or *Klebsiella* before and after the COVID-19 pandemic. By identifying

key differences and trends, we hope to contribute to a better understanding of how the pandemic has impacted the epidemiology and clinical outcomes of these infections.

### MATERIALS and METHODS

**Study Design**

Patients who were admitted to the chest diseases ICU due to hypercapnic or hypoxic respiratory failure between October 2018-April 2020 and December 2022-December 2024 were included in our study. Data on past hospitalizations and antibiotic treat- ment duration were obtained from our hospital auto- mation system and the Ministry of Health e-Nabız system. Ethics approval was obtained from the local ethics committee before initiating the study.

### Study Population

A total of 1.187 patients were monitored in our ICU during the study period. The following patients were excluded from the study:

- Patients with pneumonia at the time of ICU admission,
- Patients who acquired pneumonia while on inva- sive mechanical ventilation,
- Patients with resistant microorganism growth in respiratory tract isolates within the last six months,
- Patients who developed pneumonia within 48 hours of being connected to or disconnected from the ventilator,
- Patients with previous healthcare-associated pneumonia,
- Patients with concurrent infections (e.g., central catheter infections, urinary tract infections, or gastrointestinal system infections) alongside VAP,
- Patients who died due to causes other than pneu- monia after being diagnosed with VAP.

VAP-related mortality was defined as death occurring within 14 days after initiating specific antibiotic therapy in patients with radiologically confirmed VAP. Patients who survived beyond 14 days after tar- geted antibiotic therapy were not considered to have VAP-related mortality. Patients who completed their treatment in the hospital after VAP were included in our surveillance group.

After applying the exclusion criteria, 72 VAP patients with *A. baumannii* and/or *K. pneumoniae* growth in tracheal aspirate cultures were included in the study. To assess the impact of the COVID-19 pandemic, VAP cases were categorized into two periods: Pre- pandemic and post-pandemic. Thirty patients were identified in the pre-pandemic period, while 42 patients were identified in the post-pandemic period, following the exclusion criteria.

During the period when VAP was diagnosed, empiri- cal antibiotic therapy was initiated until culture results were obtained. Once the culture and antibio- gram results were available, treatment regimens were adjusted accordingly. Hematological and biochemi- cal parameters were recorded on the day of culture positivity and on the third day after initiating targeted treatment. Patients were monitored until ICU dis- charge or completion of hospital treatment.

### Taking a Sputum Sample

Empirical antibiotic therapy for sputum analysis was initiated only after obtaining the sample. For tracheal aspirate sampling, aspiration was performed using:

- A 50-cc sterile disposable irrigation syringe con- nected to the outer end of the intubation tube, or
- A disposable lavage tube attached to the endotra- cheal tube.

The aspirate sample was immediately sealed with its original cap and transported to the microbiology laboratory. Samples not processed within 48 hours were stored at 4°C and transported on wet ice.

A high-quality sputum sample was defined as one with <10 squamous epithelial cells per field at 10x magnification and a polymorphonuclear leukocyte count >25. Suitable sputum samples were cultured on blood, EMB, and chocolate agar media. Cultures were incubated at 37°C for 24-48 hours for bacterial growth evaluation.

Bacterial identification and antibiotic susceptibility testing were performed using the BD PHOENIX 100 automated system (Becton-Dickinson, USA), with antibiotic resistance patterns evaluated according to EUCAST criteria.

- Multi-drug resistance (MDR) was defined as resistance to at least one antibiotic in three or more antibiotic groups.

- Extensively drug-resistant (XDR) strains were defined as resistant to all antibiotics except for two or fewer antibiotic groups.

### Echocardiographic Evaluation

Echocardiographic (ECHO) examinations were con- ducted by three cardiologists using a Toshiba S270-A device equipped with a 2.5 MHz probe. The proce- dure was performed in a quiet environment while the patient was calm, positioned in the left lateral decubitus position, and breathing comfortably. Before ECHO examination, cardiac auscultation, electrocardiography, and chest radiography were performed for all patients to ensure comprehensive cardiac assessment.

During ECHO examination, multiple imaging planes were utilized, including the parasternal long-axis view, the aorto-mitral-papillary muscle view, and the parasternal short-axis view at the apical level. Additionally, apical four-chamber and two-chamber images were obtained using two-dimensional, M-mode, color Doppler, pulse Doppler, and contin- uous Doppler modalities. When necessary, the interatrial septum was evaluated using subcostal ECHO images, while the aortic arch was assessed with suprasternal ECHO imaging.

Transthoracic echocardiography was used to evalu- ate right heart hemodynamics, particularly systolic pulmonary artery pressure (SPAP). SPAP was esti- mated based on the measurement of maximum tri- cuspid regurgitation velocity, the peak pressure gra- dient of tricuspid regurgitation, and an assumed fixed value of 10 mmHg for right atrial pressure. ECHO findings were integrated into the study to explore potential correlations between cardiovascu- lar function and mortality in patients diagnosed with VAP.

### Statistical Analysis

Statistical analyses were performed using IBM SPSS Statistics for Windows version 27.0 (IBM Corp., Armonk, NY). Between-group comparisons were performed using Pearson’s chi-square test for para- metric data and Mann-Whitney U test for non-nor- mally distributed numerical data. The groups’ demo- graphic data and laboratory parameters were com- pared using Kruskal Wallis test. Independent sample t-test was used to compare the parameters for which significant results were detected with the Kruskal Wallis test between groups. Multivariate logistic

regression analysis was applied to calculate the fac- tors affecting mortality. A p< 0.05 was considered statistically significant.

## RESULTS

Mean age of the patients included in our study was

69.3 ± 12.9 years. While the mean age of the patients included in the study during the pre- pandemic period was 71.5 ± 13.3 years, it was 67.7

± 12.6 years in the post-pandemic period. No statistically significant difference was observed between the mean ages of the groups (p= 0.23). Forty-two (58.3%) of the patients included in our study were male. No statistically significant difference was observed in the analysis of the groups according to sex (p= 0.09).

Of the patients included in our study, 65 (90.3%) had chronic obstructive pulmonary disease, 38 (52.7%) had hypertension, 16 (22.2%) had diabetes

mellitus, 12 (16.7%) had coronary artery disease, 16

(22.2%) had Alzheimer’s dementia, and 10 (13.9%) were receiving immunosuppressive treatment due to malignancy or rheumatologic diseases.

While *K. pneumoniae* growth was observed along- side *A. baumannii* in 4 out of 30 patients (13.3%) in the pre-pandemic period, it was noted in 16 out of 42 patients (38.1%) in the post-pandemic period. In the comparison made before and after the pan- demic, an increase in the percentage of *Klebsiella* growth was observed after the pandemic. It was also determined that the XDR rate increased in the post- pandemic period (p= 0.009, <0.001 respectively)

(Table 1).

A comparison of age, laboratory parameters, and ECHO findings on the day growth was observed (first day of specific treatment) and on the third day of specific treatment among all patients with and with- out mortality in the study is shown in Tables 2 and 3. Accordingly, it was observed that age, white blood cell count, and neutrophil-to-lymphocyte ratio (NLR) were higher in the group that experienced mortality on the day specific treatment was started (p= 0.04,

<0.001, 0.03, respectively). However, ejection frac- tion was lower in patients who experienced mortal- ity (p= 0.03). It was observed that blood urea nitro- gen (BUN), C-reactive protein (CRP), procalcitonin, CRP/albumin ratio, and BUN levels were statistically significantly higher in cases that experienced mortal- ity on the third day of treatment (p= <0.001 for all).

**Table:** 

**Table 1.** Demographic characteristics of the patients included in the study during the pre- and post-pandemic periods, bacteria grown in tracheal aspirate, and resistance levels			
	**Before the Pandemic (n= 30)**	**After the Pandemic (n= 42)**	**p**
Age (year)	71.5 ± 13.4	67.6 ± 12.6	0.23
Sex (n/%)			
Male	14 (33.3%)	28 (66.7%)	
			0.09
Female	16 (53.3%)	14 (46.7%)	
Comorbidities (n)			
Hypertension	20	18	0.76
Diabetes mellitus	9	7	0.8
COPD	27	38	0.65
CAD	6	6	N/A
Alzheimer-dementia	10	6	0.6
Immunosuppression	6	4	0.82
*A. baumannii* (n/%)	30 (100%)	40 (95.2%)	
			0.009
*K. pneumoniae* (n/%)	4 (13.3%)	18 (42.9%)	
Drug Resistance (n/%)			
MDR	24 (80%)	8 (19%)	
			<0.001
XDR	6 (20%)	34 (81%)	
COPD: Chronic obstructive pulmonary diseases, CAD: Coronary arterial diseases, *A. baumannii*: *Acinetobacter baumannii*, *K. pneumoniae*: *Klebsiella pneumoniae*, MDR: Multi-drug resistance, XDR: Extensively-drug resistant.			

**Table:** 

**Table 2.** Comparison of laboratory parameters and echocardiography findings between the groups with and without mortality on the specific day treatment was started according to culture growth			
	**Mean ± SD Survey n= 24**	**Mean ± SD Mortality n= 48**	**p**
Age	64.7 ± 13.6	71.5 ± 12.1	0.04
WBC (/µL)	9879.2 ± 4506.9	12525.4 ± 6496.1	<0.001
Neuthrophil (/µL)	8643.3 ± 4270.1	12884.2 ± 6516.3	0.005
Lymphocyte (/µL)	780.8 ± 460.7	797.9 ± 444.6	0.8
Eosinophil (/µL)	52.5 ± 86.9	67.1 ± 138.5	0.63
Basophil (/µL)	14.2 ± 20.2	20.8 ± 21.4	0.2
Platelet (/µL)	260750 ± 72584.7	247041.7 ± 140250.7	0.65
NLR	14.3 ± 8.4	24.4 ± 21.5	0.03
PLR	521.5 ± 555.8	404.2 ± 304.2	0.34
AST (U/L)	42.4 ± 37.1	58.8 ± 150.3	0.48
ALT (U/L)	41.1 ± 37.3	35.1 ± 30.2	0.53
GGT (U/L)	69.7 ± 56.1	78.9 ± 93.1	0.6
ALP (U/L)	101.6 ± 46.3	93.1 ± 46.9	0.47
BUN (mg/dL)	33.8 ± 24.7	41.8 ± 24.2	0.2
Creatinine (mg/dL)	0.7 ± 0.5	0.9 ± 0.8	0.14
Total protein (g/dL)	5.5 ± 0.6	5.3 ± 0.6	0.41
Albumine (g/dL)	2.5 ± 0.4	2.4 ± 0.3	0.28
CRP (mg/L)	125.4 ± 106.8	143.4 ±102.7	0.49
Procalcitonin (ng/mL)	0.9 ± 1.4	1.9 ± 1.9	0.45

**Table:** 

**Table 2.** Comparison of laboratory parameters and echocardiography findings between the groups with and without mortality on the specific day treatment was started according to culture growth (continue)			
	**Mean ± SD Survey n= 24**	**Mean ± SD Mortality n= 48**	**p**
CRP/Albumine ratio	52.5 ± 52.7	62.2 ± 48.6	0.45
EF (%)	55.5 ± 1.7	52.9 ± 5.8	0.03
sPAP (mm_Hg)	41.4 ± 13.1	40.6 ± 11.4	0.8
SD: Standard deviation, WBC: White blood cells, NLR: Neuthrophil/lymphocyte ratio, PLR: Platelet/lymphocyte ratio, AST: Aspartat aminotransferase, ALT: Alanine aminotransferase, GGT: Gamma glutamyl transferase, ALP: Alkaline phosphatase, BUN: Blood urea nitrogen, CRP: C-reactive protein, EF: Ejection fraction, sPAP: Systolic pulmonary arterial pressure.			

**Table:** 

**Table 3.** Comparison of laboratory parameters on the third day of treatment between the groups with and without mortality			
	**Mean ± SD Survey n= 24**	**Mean ± SD Mortality n= 48**	**p**
WBC (/µL)	10651.6 ± 4239.5	14225.3 ± 7674.3	0.04
Neuthrophil (/µL)	87890 ±3735.6	12530 ± 7775.6	0.03
Lymphocyte (/µL)	1216.6 ± 574.4	1057.4 ± 698.8	0.35
Eosinophil (/µL)	109.2 ± 140.5	77.4 ± 123.5	0.35
Basophil (/µL)	10.8 ± 11.4	16.3 ± 14.8	0.1
Platelet (/µL)	231333.3 ± 124082.3	212842.1 ± 124443.1	0.57
NLR	8.9 ± 5.2	18.2 ± 16.9	0.01
PLR	257.9 ± 269.4	253.3 ± 140.4	0.93
AST (U/L)	38.3 ± 33.4	67.4 ± 146.1	0.34
ALT (U/L)	50.8 ± 70.9	53.1 ± 124.5	0.9
GGT (U/L)	73.8 ± 65.1	53.4 ± 41.1	0.14
ALP (U/L)	103.4 ± 42.9	95.5 ± 53.9	0.52
BUN (mg/dL)	28.1 ± 11.1	48.2 ± 32.5	<0.001
Creatinine (mg/dL)	0.6 ± 0.2	0.9 ± 0.9	0.02
Total protein (g/dL)	5.6 ± 0.6	5.2 ± 0.8	0.02
Albumine (g/dL)	2.6 ± 0.3	2.3 ± 0.3	<0.001
CRP (mg/L)	79.4 ± 69.2	163.5 ± 78.4	<0.001
Procalcitonin (ng/mL)	0.3 ± 0.5	8.4 ± 2.3	<0.001
CRP/Albumine ratio	33.2 ± 30.2	72.8 ± 37.3	<0.001
SD: Standard deviation, WBC: White blood cells, NLR: Neuthrophil/lymphocyte ratio, PLR: Platelet/lymphocyte ratio, AST: Aspartat aminotransferase, ALT: Alanine aminotransferase, GGT: Gamma glutamyl transferase, ALP: Alkaline phosphatase, BUN: Blood urea nitrogen, CRP: C-reactive protein.

The comparison of antibiotic susceptibility rates is given in Table 4. Accordingly, a statistically signifi- cant decrease in susceptibility to carbapenems was observed along with beta-lactam antibiotics (p< 0.001). Although there was a decrease in susceptibil- ity to ceftazidime-avibactam, the most sensitive anti- biotic for *K. pneumoniae*, it was noted that there was no statistically significant difference (p= 0.09). While the number of patients with XDR in the pre-pandem- ic period was 6 (20%), this number increased to 34 (81%) after the pandemic. A statistically significant

difference was observed in the comparison (p< 0.001).

Regression analysis of age, comorbidities, ECHO findings, drug resistance status (MDR or XDR), the number of hospitalizations in the last year, and the number of days of antibiotic use in patients who developed mortality during follow-up are shown in Table 5. Accordingly, it was observed that advanced age, the presence of coronary artery disease, a low ejection fraction, and XDR drug resistance increased mortality (p= 0.03, 0.04, 0.04, 0.001, respectively).

**Table:** 

**Table 4.** Drug susceptibility rates of *A. baumannii* and *K. pneumoniae* in the pre-pandemic and post-pandemic periods					
	**pre-Pandemic Sensitivity (%)**		**post-Pandemic Sensitivity (%)**		
	***A. baumannii*** **(n= 30)**	***K. pneumoniae*** **(n= 4)**	***A. baumannii*** **(n= 40)**	***K. pneumoniae*** **(n= 18)**	**p**
Imipenem	80	25	12.5	27.7	<0.001
Colistin	100	-	100	-	N/A
Levofloxacin	13.3	25	12.5	16.6	0.87
Cefazolin	-	0	-	0	N/A
Cefepime	-	25	-	11.1	0.03
Cefoxitin	-	50	-	22.2	<0.001
Ceftazidime	16.6	25	15	5.5	0.02
Ticarcillin-clavulanic acid	-	0	-	0	N/A
Ceftazidime-avibactam	-	100	-	83.3	0.09
TMP/SMX	80	50	20	11.1	<0.001
Piperacillin	-	50	-	50	N/A
Ceftriaxone	-	25	-	0	<0.001
Amoxicillin-clavulanate	-	25	-	0	<0.001
Amikacin	-	50	-	5.5	<0.001
Ampicillin-sulbactam	-	25	-	0	<0.001
Piperacillin-tazobactam	0	25	0	5.5	<0.001
Cefoperazone	0	50	0	50	N/A
Cefotaxime	0	25	0	5.5	<0.001
Gentamicin	-	25	-	11.1	0.01
Meropenem	80	50	15	11.1	<0.001
Cefuroxime	-	0	0	0	N/A
Ciprofloxacin	0	0	0	0	N/A
*A. baumannii*: *Acinetobacter baumannii*, *K. pneumoniae*: *Klebsiella pneumoniae,* TMP/SMX: Trimethoprim/sulfamethoxazole. p: Comparison of total antibiotic susceptibility rates before and after the pandemic.					

**Table:** 

**Table 5.** Multivariate regression analysis of the effects of age, comorbidities, echocardiographic findings, drug resistance, the number of hospitalizations in the last year, and the duration of antibiotic use on mortality								
	**B**	**S.E.**	**Wald**	**df**	**p**	**EXP(B)**	**95% CI for EXP(B)**	
							**Lower**	**Upper**
Age (year)	0.10	0.05	4.49	1	0.03	1.11	1.01	1.22
COPD	0.52	1.15	0.21	1	0.65	1.68	0.17	16.07
Diabetes mellitus	0.19	1.33	0.02	1	0.89	1.2	0.09	16.34
CAD	3.28	1.57	4.37	1	0.04	26.56	1.23	575.16
Hypertension	1.33	0.93	2.04	1	0.15	0.26	0.04	1.64
Immunosuppression	-2.07	1.2	2.97	1	0.09	0.13	0.01	1.33
EF (%)	-0.29	0.15	4.03	1	0.04	0.75	0.56	0.99
sPAB (mm_Hg)	-0.03	0.04	0.55	1	0.46	0.97	0.91	1.04
Drug resistance	-4.31	1.34	10.35	1	0.001	0.01	0.001	0.19
Number of hospitalizations in the last year	0.79	0.87	0.84	1	0.36	2.21	0.41	12.03
Duration of antibiotic use in the past year	-0.16	0.12	1.68	1	0.19	0.85	0.67	1.08
CI: Confidence interval, COPD: Chronic obstructive pulmonary diseases, CAD: Coronary arterial diseases, EF: Ejection fraction, sPAP: Systolic pul- monary arterial pressure.								

## DISCUSSION

In our study evaluating the ICU dynamics before and after the COVID-19 pandemic, we observed a significant increase in XDR *K. pneumoniae* cases. Among patients with *A. baumannii* and/or *K. pneumoniae* detected in tracheal aspirates, the primary cause of mortality was antibiotic resistance, followed by advanced age, low ejection fraction, and immunosuppression. Additionally, we found that elevated CRP/albumin and NLR levels on the third day of targeted antibiotic therapy could serve as potential indicators of mortality risk.

The recent increase in antibiotic resistance has caused significant increases in hospital stays. In addi- tion, MDR and XDR infections due to gram-negative agents are among the most important causes of mor- tality in ICUs (8). It has been reported that nearly five million people worldwide lost their lives due to MDR infections in 2019 (9). *A. baumannii* and *K. pneumo- niae* were the most frequently isolated gram-negative agents in HAP infections observed in ICUs (10). In the study conducted by Durdu et al., the rate of *Klebsiella* isolates with MDR was 73%, while the rate of XDR was 14% (11). In the study conducted on the resistance rate in *A. baumannii* strains observed in ICUs in our country, the XDR rate was 71.6% (9). In our study, when evaluating both *A. baumannii* and *K. pneumoniae* strains together, we found that the XDR rate before the pandemic was significantly lower than previously reported national data. However, this rate dramatically increased after the pandemic, rising from 20% to 81%. This stark increase highlights the profound impact of the COVID-19 pandemic on anti- microbial resistance patterns in the ICU setting.

During and after the COVID-19 pandemic, no effec- tive antiviral treatment was available for the virus itself. Consequently, high-dose corticosteroid therapy and anti-cytokine treatments were widely adminis- tered to manage severe cases. However, these inter- ventions facilitated the development of secondary bacterial infections, particularly among critically ill patients. Despite global warnings against the indis- criminate use of antibiotics, effective antimicrobial stewardship programs remained insufficient (12,13). Our ICU, which we included in the study, was used by the chest diseases clinic before the pandemic. However, during the pandemic, it was transformed into an ICU that only accommodated COVID-19 patients. Although the ICU was transferred back to

our clinic after the pandemic, it became painfully clear that some things were not the same. The most critical situation that caught our attention at first was the resistance to carbapenems, which are widely used in COVID-19 patients. However, it was later determined that the same situation existed in XDR and MDR *Acinetobacter* and *Klebsiella* species.

In our study, we observed an increase in XDR *Acinetobacter* and *Klebsiella* in our ICU and the fre- quency of these agents in patients who developed VAP. This situation was evaluated as compatible, especially compared to studies conducted during and after the pandemic (14-17). However, the rate of XDR gram-negative pathogens was higher in our ICU. In the laboratory analysis performed on the third day of specific treatment, it was observed that the acute phase reactant and BUN levels were higher in cases with mortality. This situation may have devel- oped due to a lack of response to specific treatment and impaired tissue perfusion in cases with mortality. Carbapenem susceptibility has decreased signifi- cantly in resistant *Klebsiella* strains. Ceftazidime- avibactam has been determined to be the most reli- able treatment we can currently use. Studies have also reported a significant decrease in *Klebsiella* growth after ceftazidime-avibactam (18). In the mul- tivariate regression analysis of mortality risk factors in VAP patients, increased antimicrobial resistance was identified as a more critical determinant of mortality than coronary artery disease, low ejection fraction, or advanced age. This finding reinforces the urgent need for enhanced antimicrobial stewardship programs to mitigate ICU mortality driven by drug resistance.

Our study focused on evaluating only one ICU before and after the pandemic, and the results need to be strengthened with studies that evaluate data from many ICU together. However, the only ICU in our hospital where the COVID-19 impact was most clearly observed was the chest diseases ICU, and we aimed to show the unnecessary antibiotic effect clearly.

Antibiotic resistance, particularly the rise of MDR and XDR *Acinetobacter* and *Klebsiella* strains, is creating a silent pandemic in ICUs worldwide. If urgent measures are not taken, the increasing prevalence of drug-resistant infections will lead to uncontrollable consequences. Without immediate global and national interventions, the mortality burden of HAP will continue to rise exponentially.

**Ethical Committee Approval:** This study was approved by Erzurum University Faculty of Medicine Clinical Research Committee (Decision no: BAEK 2024/03-59 Date: 13.03.2024).

### CONFLICT of INTEREST

The authors declare that they have no conflict of interest.

## AUTHORSHIP CONTRIBUTIONS

Concept/Design: BK, FK Analys/Interpretation: KÇ, GK Data acqusition: KÇ, GK Writing: BK, FK

Clinical Revision: FK Final Approval: BK, FK

## REFERENCES

1. *Klompas M, Branson R, Cawcutt K, Crist M, Eichenwald EC, Greene LR, et al. Strategies to prevent ventilator-asso- ciated pneumonia, ventilator-associated events, and non- ventilator hospital-acquired pneumonia in acute-care hospitals: 2022 Update. Infect Control Hosp Epidemiol 2022; 43(6): 687-713. https://doi.org/10.1017/ice.2022.88*
2. *Miron M, Blaj M, Ristescu AI, Iosep G, Avădanei A-N, Iosep D-G, et al. Hospital-acquired pneumonia and venti- lator-associated pneumonia: A literature review. Microorganisms 2024; 12(1): 213. https://doi.org/10.3390/microorganisms12010213*
3. * Kinross P, Gagliotti C, Merk H, Plachouras D, Monnet DL, Högberg LD, et al. Large increase in bloodstream infec- tions with carbapenem-resistant acinetobacter species during the first 2 years of the COVID-19 pandemic, EU/ EEA, 2020 and 2021. Euro Surveill 2022; 27(46):2200845. https://doi.org/10.2807/1560-7917.ES.2022.27.46.2200845*
4. * Wicky PH, Niedermann MS, Timsit JF. Ventilator-associated pneumonia in the era of COVID-19 pandemic: How com- mon and what is the impact? Critical Care 2021; 25(1):153. https://doi.org/10.1186/s13054-021-03571-z*
5. *Abubakar U, Al-Anazi M, Rodríguez-Baño J. Impact of COVID-19 pandemic on multidrug resistant gram positive and gram negative pathogens: A systematic review. J Infect Public Health 2023; 16(3): 320-31. https://doi.org/10.1016/j.jiph.2022.12.022*
6. *Salehi M, Jafari S, Ghafouri L, Ardakani HM, Abdollahi A, Beigmohammadi MT, et al. Ventilator-associated pneumo- nia: Multidrug resistant acinetobacter vs. extended spec- trum beta lactamase-producing Klebsiella. J Infect Dev Ctries 2020; 14(06): 660-3. https://doi.org/10.3855/jidc.12889*
7. *Liu CW, Chen Q, Ding N, Hu LF. Ceftazidime-avibactam: Combination therapy versus monotherapy in the chal- lenge of pneumonia caused by carbapenem-resistant Klebsiella pneumoniae. Heliyon 2024; 10(16): e35757. https://doi.org/10.1016/j.heliyon.2024.e35757*
8. *Diao H, Lu G, Zhang Y, Wang Z, Liu X, Ma Q, et al. Risk factors for multidrug-resistant and extensively drug-resis- tant acinetobacter baumannii infection of patients admit- ted in ICU: A systematic review and meta-analysis. J Hosp Infect 2024; 149: 77-87. https://doi.org/10.1016/j.jhin.2024.04.013*
9. *Uluç K, Kutbay Özçelik H, Akkütük Öngel E, Hırçın Cenger D, Çolakoğlu ŞM, Köylü Ilkaya N, et al. The preva- lence of multidrug-resistant and extensively drug-resistant infections in respiratory intensive care unit, causative microorganisms and mortality. Infect Drug Resist. 2024; 17: 4913-9. https://doi.org/10.2147/IDR.S480829*
10. *Sim JK, Min KH, Yoo KH, Jeon K, Chang Y, Hong SB, et al. Impact of multidrug-resistant bacterial colonization on clinical characteristics, antibiotic treatment, and clinical outcomes of hospital-acquired pneumonia. Lung 2025; 203(1): 17. https://doi.org/10.1007/s00408-024-00762-7*
11. *Durdu B, Kritsotakis EI, Lee AC, Torun P, Hakyemez IN, Gultepe B, et al. Temporal trends and patterns in antimi- crobial-resistant Gram-negative bacteria implicated in intensive care unit-acquired infections: A cohort-based surveillance study in Istanbul, Turkey. J Glob Antimicrob Resist 2018; 14: 190-6. https://doi.org/10.1016/j.jgar.2018.04.015*
12. *Debaco ISS, Kluck HM, Marx R, Rosa PRMd, Teixeira C. Bacterial coinfections in COVID-19-hospitalized patients. Revista da Associação Médica Brasileira. 2024; 70(2): e20230469. https://doi.org/10.1590/1806-9282.2023469*
13. * Patton MJ, Gaggar A, Might M, Erdmann N, Orihuela CJ, Harrod KS. Community-acquired bacterial coinfections and COVID-19. Physiological Reviews 2024; 104(1):1-21. https://doi.org/10.1152/physrev.00010.2023*
14. *Moretti M, Deyi VYM, De Geyter D, Wybo I, Claus M, Jonckheer J, et al. The impact of the COVID-19 pandemic on gram-negative bacteria susceptibility patterns in respi- ratory samples of intensive care units in the Brussels Capital Region, 2010-2021. Am J Infect Control 2024; 52(3):305-11.https://doi.org/10.1016/j.ajic.2023.08.020*
15. *Spiliopoulou A, Giannopoulou I, Assimakopoulos SF, Jelastopulu E, Bartzavali C, Marangos M, et al. Laboratory surveillance of acinetobacter spp. bloodstream infections in a tertiary university hospital during a 9-year period. Trop Med Infect Dis 2023; 8(11): 503. https://doi.org/10.3390/tropicalmed8110503*
16. *Abbas S, Sabir AU, Khalid N, Sabir S, Khalid S, Haseeb S, et al. Frequency of extensively drug-resistant gram-nega- tive pathogens in a tertiary care hospital in Pakistan. Cureus 2020; 12(12): e11914. https://doi.org/10.7759/cureus.11914*
17. *Lagadinou M, Amerali M, Michailides C, Chondroleou A, Skintzi K, Spiliopoulou A, et al. Antibiotic resistance trends in carbapenem-resistant gram-negative pathogens and eight-year surveillance of XDR bloodstream infections in a Western Greece Tertiary Hospital. Pathogens 2024; 13(12): 1136. https://doi.org/10.3390/pathogens13121136*
18. *Vidal Cortés P, Martin Loeches I, Rodríguez A, Bou G, Cantón R, Diaz E, et al. Current positioning against severe infections due to Klebsiella pneumoniae in hospitalized adults. Antibiotics 2022; 11(9): 1160. https://doi.org/10.3390/antibiotics11091160*

